# Closing the Hispanic/Latino engagement gap in diabetes prevention: feasibility of a community co-designed, participate-to-lead program

**DOI:** 10.1093/tbm/ibag054

**Published:** 2026-08-22

**Authors:** Maribel Cedillo, Jesell Zepeda, Chaorong Wu, Isabel Teresa Molina, Molly B Conroy

**Affiliations:** Division of General Internal Medicine, University of Utah, Salt Lake City, UT, United States; Division of General Internal Medicine, University of Utah, Salt Lake City, UT, United States; Division of General Internal Medicine, University of Utah, Salt Lake City, UT, United States; Division of Epidemiology, Department of Internal Medicine, University of Utah, Salt Lake City, UT, United States; University Neighborhood Partners, School of Social Work, University of Utah, Salt Lake City, UT, United States; College of Social Work, University of Utah, Salt Lake City, UT, United States; Division of General Internal Medicine, University of Utah, Salt Lake City, UT, United States; Department of Population Health Science, University of Utah, Salt Lake City, UT, United States

**Keywords:** diabetes prevention, Hispanic Americans, health disparities, feasibility studies, patient engagement, community-based participatory research, Hispanic/Latino, culturally relevant interventions, model of culture

## Abstract

**Background:**

Hispanic/Latino (H/L) adults show lower engagement and smaller weight-loss outcomes than non-Hispanic White (NHW) participants in the National Diabetes Prevention Program (NDPP), a gap driven by delivery rather than biological differences.

**Purpose:**

To evaluate the feasibility, engagement, and preliminary clinical outcomes of *Paso a Paso*, a community co-designed, culturally relevant diabetes prevention program for Spanish-speaking H/L adults, across two cohorts: instructor-trainees (Cohort 1) and community participants (Cohort 2).

**Methods:**

Single-arm feasibility pilot guided by RE-AIM, EPIS, and ERIC frameworks. Cohort 1 (*n* = 18 H/L community leaders) completed 16 weekly in-person sessions; Cohort 2 (*n* = 30 at two sites) completed 13–15 weekly sessions, both led by a registered dietitian. Participants were classified into three engagement tiers: high-dose (≥50% sessions), low-dose community-connected (<50% but retained at the final session), and discontinued. Body mass index (BMI), waist circumference, and HbA1c were assessed at baseline and 4 months.

**Results:**

Cohort 1 achieved 100% retention and a median attendance rate of 93.8% (range 31.2%–100.0%). Cohort 2 achieved 73% 4-month retention and a median attendance rate of 61.5% (range 0.0%–93.3%). Among 21 paired participants, mean % body weight change was −1.5% (SD, 2.3) and 81% achieved any weight reduction; participants with overweight and obesity, the primary target population, lost −1.7% (SD, 1.5) and −1.9% (SD, 2.6) respectively.

**Conclusions:**

*Paso a Paso* suggests feasibility, with engagement and retention exceeding NDPP benchmarks for H/L participants and preliminary, clinically meaningful weight reductions observed across the winter holiday season. These hypothesis-generating findings support advancement to a powered hybrid effectiveness-implementation trial.

## Graphical abstract

**Graphical Abstract:**
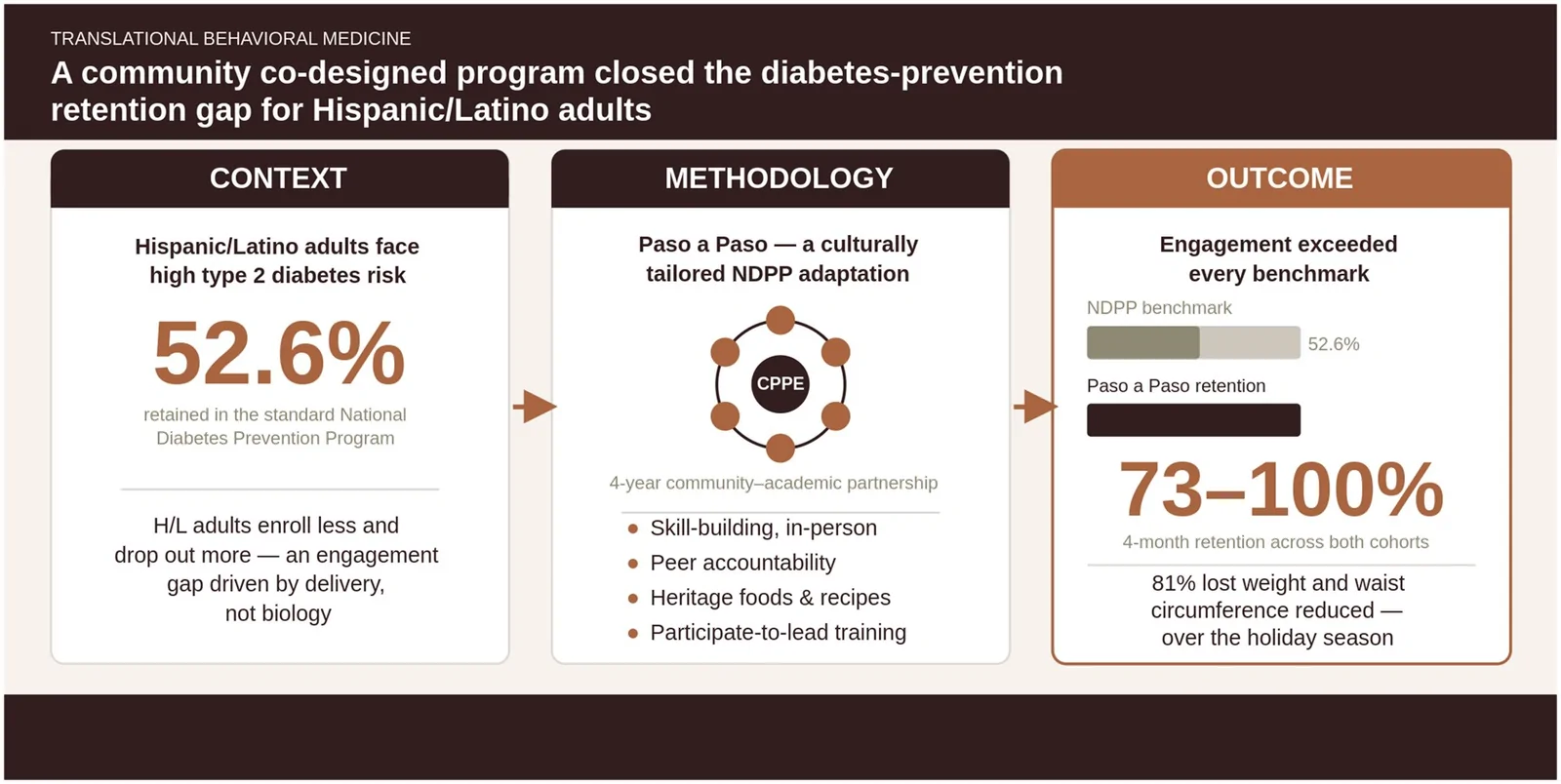

## Teaser

A community co-designed diabetes prevention program for Spanish-speaking Hispanic/Latino adults achieved attendance and retention exceeding published NDPP benchmarks for this population, suggesting culturally tailored, community-led delivery may close long-standing engagement gap.

Implications

**Researchers:** Advance community co-designed, culturally relevant weight management and diabetes prevention programs to a powered hybrid type 1 effectiveness-implementation trial, pairing weight and HbA1c as effectiveness endpoints with engagement tier classification as an implementation endpoint in Spanish-speaking Hispanic/Latino adults.
**Practitioners:** Adopt a participate-to-lead coach training model, in which community leaders complete the full program before delivering it, and structure delivery around community sites, trusted relationships, and cultural fidelity to close Hispanic/Latino engagement gaps that standard approaches leave unaddressed.
**Policymakers:** Expand CDC DPRP recognition and Medicare MDPP reimbursement pathways to explicitly support community-based delivery by culturally matched, language-concordant staff, alongside a registered dietitian.

## Introduction

The Hispanic/Latino (H/L) population in the United States has surpassed 65 million [1] and faces a disproportionate burden of Type 2 diabetes (T2D). Approximately 20–24 million H/L adults have prediabetes. H/L adults develop T2D up to 7 years earlier than non-Hispanic Whites (NHW) [2] and at lower BMI [3]. Although numerous risk factors contribute, including genetic predisposition [4, 5], lifestyle [4, 5], acculturation [4, 6], and social determinants of health [7], obesity is the primary driver of elevated T2D risk [4, 8]. Alarmingly, over 77% of H/L adults are classified as overweight and approximately 45% meet criteria for obesity rates that exceed those of NHW adults; more than half are projected to develop T2D during their lifetime [9–11]. As the H/L population rises toward 28% of U.S. residents by 2060 [12], urgent action is needed.

Randomized controlled trials have consistently demonstrated that modest, intentional weight loss of 5%–7% reduces T2D incidence by up to 58% [13–16], primarily through improvements in insulin sensitivity and preservation of β-cell function. The National Diabetes Prevention Program (NDPP) [17], a CDC-supported translation of this evidence, represents the cornerstone of U.S. diabetes prevention efforts. Despite its broad dissemination, clinically meaningful outcomes, particularly achieving ≥5% weight loss, are highly dependent on sustained participant engagement [18, 19] and substantial disparities persist in NDPP outcomes [19–22]. H/L participants are significantly less likely than NHW participants to attend sessions, complete the core curriculum, or achieve clinically meaningful weight loss [20], and they consistently demonstrate the lowest retention rates [20–22]. Notably, these disparities appear to be driven largely by nonbiological factors: when attendance is accounted for, weight loss outcomes are comparable across ethnic groups [20].

Prior efforts to close this gap have relied largely on culturally and linguistically adapted versions of the NDPP [17], principally the Spanish-language NDPP (S-NDPP) [23–25] and other surface-level tailoring delivered in a top-down manner. These adaptations did not address the structural engagement barriers (trust, program relevance, community ownership, and competing demands) that drive H/L attrition, and disparities persisted even with Spanish-language delivery. Direct evidence underscores this ceiling: low-income H/L participants remained less likely to enroll [26], more likely to drop out, and less likely to reach 5% weight loss even when program cost was eliminated and materials were culturally adapted [27, 28], while H/L enrollees show higher no-show rates and lower first-session attendance than NHW participants [29], built-environment, financial, and social-alienation barriers are structurally unaddressed by the standardized group format [30], and low engagers report limited diagnosis awareness, skepticism about program relevance, and a perception that participation burden outweighs benefit [31, 32]. Moreover, prior culturally tailored DPP adaptations have been only modestly effective: a systematic review of 12 such studies found limited weight loss and little documentation of rigorous participatory processes or cultural concepts (on average one concept, most often familismo), with most being small trials against usual-care controls and only one compared against the S-NDPP [33]. Even language adaptation shows limitations, clinically meaningful (≥5%) weight loss was achieved by only 16% of H/L participants in the standard NDPP and 19% in the S-NDPP, versus 26% in NHW participants [20]. These persistent disparities are therefore driven by unaddressed structural and cultural-fit barriers rather than by language alone, establishing the premise for a community co-designed approach purpose-built to improve engagement.

To address these engagement-related disparities, culturally and contextually tailored interventions that target known barriers to participation are needed. *Paso a Paso* was developed as an adaptation of the NDPP designed specifically to improve engagement, retention, and weight-loss outcomes among H/L adults at risk for T2D. Over four years, our interdisciplinary community–academic partnership (CAP) [34, 35] collaborated with H/L residents and community-based organizations in Salt Lake County, Utah to adapt the Spanish-language NDPP to better align with community priorities, local context, and lived experiences. Using a Comprehensive Participatory Planning and Evaluation (CPPE) approach, community members identified trust, practical skill-building, and respect for cultural heritage as essential elements for program relevance and sustainability. Community leaders further emphasized the importance of a “participate-to-lead” model, in which future facilitators first complete the program themselves, a recommendation that directly informed the two-phase pilot design evaluated in this study [34–36].

The resulting intervention, *Paso a Paso*, is a culturally tailored lifestyle program for weight management and diabetes prevention that retains the core NDPP structure while incorporating community-driven adaptations. The program consists of 16 weekly, in-person core sessions (months 1–4), followed by eight monthly maintenance sessions delivered remotely (months 5–12), and is led by a registered dietitian nutritionist (RDN). This manuscript reports on the feasibility, engagement, and preliminary clinical outcomes of *Paso a Paso* across two cohorts: (i) Cohort 1, comprised of community leader instructor-trainees who completed the program as preparation for future facilitation, and (ii) Cohort 2, comprised of community participants enrolled at two community sites (Site A, community library [Cohort 2a] and Site B, community clinic [Cohort 2b]).

## Methods

### Study design

This single-arm feasibility pilot was guided by the RE-AIM (Reach, Effectiveness, Adoption, Implementation, Maintenance) [37] and EPIS (Exploration, Preparation, Implementation, Sustainment) frameworks [34, 35, 38]. This study was approved under University of Utah IRB Protocol No. 00183257. All procedures were carried out in accordance with the ethical standards of the institutional research committee and with the 1964 Declaration of Helsinki and its later amendments. All participants provided written informed consent in Spanish or English.

RE-AIM guided outcome specification across the five domains: Reach was operationalized as enrollment relative to eligible community members identified through CAP partner networks; Effectiveness was indexed by body weight, BMI, waist circumference, and HbA1c change; Adoption was captured at the site level across the two community delivery settings; Implementation was measured through the three-tier engagement classification and session-level attendance; and Maintenance is being assessed via the eight monthly remote sessions in months 5–12. EPIS structured the multi-year project timeline: the Exploration and Preparation phases correspond to the previous CAP co-design and CPPE process preceding this pilot [34, 35, 39, 40], the Implementation phase comprises the two cohorts reported here, and the Sustainment phase will assess whether Cohort 1 instructor-trainees deliver subsequent cohorts without direct RDN supervision.

### Participants

#### Cohort 1 (participate-to-lead)

H/L community representatives were recruited via word-of-mouth among Spanish-speaking H/L leaders and CAP partner networks and self-enrolled in person during the orientation session. Eligibility required Spanish fluency and interest in delivering community health programs; glycemic status and BMI were not eligibility criteria. Thirty community leaders attended an orientation session; 18 were formally enrolled, capped by available CTSI training capacity. Enrollment was completed within a 1- to 2-week recruitment window. All completed 16 core in-person sessions, and a 30-min post-session debrief at each session [34, 35, 39, 40]. Trainees received a $50 gift card per session to offset the time and travel costs of attending the 16 sessions, together with recognition of program completion and continued eligibility to serve as Paso a Paso instructors. Upon completing the full program, trainees became eligible instructors who co-deliver (“facilitate”) subsequent community cohorts, co-leading sessions, delivering curriculum content, and facilitating group activities, under RDN supervision and mentorship (supervised co-teaching).

The participate-to-lead implementation strategy was requested during the CPPE process and can be decomposed into four discrete components, mapped to Expert Recommendations for Implementing Change (ERIC) taxonomy terms: (i) *full-program experiential training* (ERIC: “conduct ongoing training”), in which each prospective instructor completed all 16 core sessions as a participant before any teaching role; (ii) *structured post-session debriefs* (ERIC: “facilitate relay of clinical data to providers” adapted to facilitator preparation), consisting of 30-min reflective sessions immediately following each of the 16 core sessions to process content delivery, cultural fidelity, and group dynamics; (iii) *supervised co-teaching with RDN mentorship* (ERIC: “provide clinical supervision”), in which graduate trainees co-led subsequent community cohorts alongside the RDN; and (iv) *recognition of program completion* (ERIC: develop educational materials paired with recognition), establishing trainees as eligible *Paso a Paso* instructors under continued RDN oversight.

#### Cohort 2 (community participants)

Eligibility included Spanish-speaking adults aged 18–77 years; BMI ≥25 kg/m² or a CDC Prediabetes Risk Test score ≥2 (administered by research staff). The ≥2 threshold was a deliberate, prespecified choice appropriate to a feasibility pilot whose primary aims were enrollment, and engagement, outcomes that have been persistently difficult to achieve among H/L adults, who remain markedly under-enrolled in the NDPP (only 10% of 14 747 participants in the national NDPP evaluation identified as H/L, and just 16% of Latino participants achieved ≥5% weight loss) [41]. Despite the inclusive threshold, the enrolled sample carried risk well above the cut point: the mean CDC Prediabetes Risk Test score was 4.3 in Cohort 1 and 4.0 in Cohort 2. In Cohort 2, A1C (*n* = 29), 24.1% (*n* = 7) fell within the prediabetic range (5.7%–6.4%) and 3.4% (*n* = 1) fell within the diabetic range (≥6.5%).

Exclusion criteria included current or planned pregnancy, recent or planned bariatric surgery, and edematous states. Approximately 50 community members expressed interest; 30 were enrolled across two community sites (Site A [Cohort 2a], *n* = 18; Site B [Cohort 2b], *n* = 12), capped by site group-delivery capacity and available funding. Enrollment was completed within a 1–2 week recruitment window. Recruitment occurred at the two community sites—the community library (Site A) and the community health center (Site B), through word-of-mouth outreach (including trained community coaches and CAP partners) and a posted sign-up sheet at the library site. Cohort 2 participants received no compensation or incentives for participation.

### Setting and context

All sessions were delivered at community sites in Salt Lake County, Utah. Due to a three-month delay in Clinical Translational Science Institute (CTSI) funding and the holiday season, cohorts completed fewer sessions than planned: Cohort 2a/Site A (15 sessions) and Cohort 2b/Site B (13 sessions). These totals served as denominators for all Cohort 2 attendance calculations. Maintenance sessions will be conducted remotely via videoconference.

### Intervention

*Paso a Paso* is a culturally relevant weight management and diabetes prevention intervention co-designed with H/L community members and [34, 35, 39, 40] led by a registered dietitian nutritionist (RDN). Core components include: 16 weekly 120-min sessions covering weight management and diabetes prevention; six hands-on cooking workshops; six physical activity sessions; and cultural adaptations emphasizing group trust, respect, and fidelity to community values. Cohort 2 was led by a RDN + 2 to 3 graduate instructor-trainees. The adaptations were generated bottom-up through a Comprehensive Participatory Planning and Evaluation (CPPE) process conducted under a shared-leadership community–academic partnership (Ventanillas de Salud at the Mexican Consulate, University Neighborhood Partners and affiliated CBOs, and the University of Utah). Thirteen community members (87% attendance) and 14 CBO leaders (93% attendance) participated in three Spanish-language collaborative meetings to review the S-NDPP and rate the importance, acceptability, appropriateness, and feasibility of its content [34, 35, 36, 39, 40]. Several adaptations were prioritized specifically to improve engagement and retention, including group-based experiential learning (cooking, eating, sharing recipes, and exercising together), peer-based accountability in place of solitary tracking, respect for heritage foods and culture, de-emphasis of weight-centric goals and calorie counting to reduce discouragement-driven dropout, expert-led in-session physical activity, participate-to-lead (if instructing), and Spanish-language facilitation at trusted community sites. Although community-derived rather than theory-driven, these adaptations align coherently with the validated Minkov-Hofstede two-dimensional model of culture: Individualism–Collectivism and Flexibility–Monumentalism—which the first author applied to the NDPP in her doctoral dissertation [36] and which we use here as an interpretive lens (Tables 1 and 2). Briefly, because the standard NDPP emphasizes individual self-accountability, self-monitoring, self-efficacy, individual calorie management, and individual weight-focused change, it aligns with individualistic behavior change strategies. However, strategies align poorly with the higher collectivism and monumentalism of many H/L communities. Community-identified adaptations consistently shift toward group interdependence (collectivism) and towards skill building, pride, and gradual change (monumentalism).

**Table 1:** Individualist National Diabetes Prevention Program (NDPP) [49] strategies and proposed collectivist strategies for diabetes prevention.

Individualism-Collectivism (IDV-COLL) traits [50, 51]	Individualist NDPP [49] strategies	Proposed collectivist NDPP strategies
**Individualist cultures**: loose ties between individuals, with expectations to care for themselves and their immediate families [50, 51] **Collectivist cultures**: strong, cohesive in-groups, often extended families, protect in exchange for loyalty [50, 51] Distinguishes wealthier, individualistic societies from poorer, collectivist ones **IDV-COLL** reflects differences in the degree to which the human self is restrained by and submitted to group interests, which may include privileges for the powerful and neglect of, or discrimination against, the powerless [52]	**Nutrition** Self-monitoring calories Self-monitoring grams of fat Self-monitoring body weight Personalized calorie and grams of fat goals Personalized eating plan **Physical Activity** Personalized physical activity goals Personalized planning and preparation for physical activities Self-monitoring strategies toward goals Individual physical activity performance and achievement **Behavior-change strategies** Individual cues or reminders Self-monitoring Personalized goal setting Individual planning for success Individual and incremental improvements toward goals Individual achievements positive reinforcement	**Nutrition** A hands-on and practical approach with tangible skills and experiences, including cooking together, eating together, sharing recipes **Physical Activity** Exercising as a group Physical activity during intervention meetings Group agreement for activity selection **Behavior-change strategies** Learning with and from the group through shared experiences Sharing progress with peers and instructors in a group setting Support from the group Group-based goal setting to promote shared progress and collective motivation rather than solely individual achievements Family and peer involvement by integrating family members into some sessions, reinforcing behavior change within the household Community-driven interventions utilizing community health workers or culturally relevant community leaders to enhance trust and participation Shared learning experiences, such as group cooking classes and physical activities, emphasizing social connectedness outside meeting intervention **Other considerations** Use language that stresses togetherness, community, and that better health helps to be there for family, loved ones, and community A safe place for emotional support and trust-building Longer sessions allowing time for socializing and bonding

Abbreviations: IDV–COLL = Individualism–Collectivism; NDPP = National Diabetes Prevention Program.

Adaptations were identified by the community through the Comprehensive Participatory Planning and Evaluation (CPPE) process [34, 35, 39, 40]; the framework used to organize and interpret them is from the first author’s dissertation [36].

**Table 2:** Flexibility-oriented National Diabetes Prevention Program (NDPP) [49] strategies and proposed monumentalist strategies for diabetes prevention.

Flexibility-Monumentalism (FLX-MON) [52]	Flexibility-oriented National Diabetes Prevention Strategies [49]	Monumentalist Diabetes Prevention Strategies
**Flexible** cultures emphasize adaptability, self-improvement, self-transformation, and continuous learning, which fosters innovation and openness to change [53] **Monumentalism** cultures highlight the importance of tradition, stability, social obligation, and adherence to established norms and usually resist changes [53] **FLX-MON** reflects differences in the degree to which the self-restrains its natural impulses in an attempt to achieve positive change through the acquisition of complex modern knowledge and skills [52]	**Nutrition** Mastering nutrition knowledge for healthier food choices, smaller portions, and adherence to calorie and fat goals **Physical Activity** Individual and incremental physical activity goals Mastering physical activity knowledge on benefits of physical activity for better health and calorie burning **Behavior-change strategies** Personal accountability Personal responsibility Problem-solving Self-efficacy Self-monitoring Self-weighing Reflecting on personal progress Personalized planning Independent learning through lessons, assignments, and homework	Respect for cultural identity by framing diabetes prevention as a means of preserving family traditions and ensuring generational well-being Language that promotes pride in taking care of the family’s well-being and health Language that promotes pride for being an example of a healthy lifestyle for family members and the community Pride and respect for heritage foods, dishes, and culture Physical activity during the sessions led by respected experts Culturally adapted nutrition education that incorporates traditional foods and dietary patterns rather than imposing unfamiliar dietary restrictions Structured learning with expert guidance that aligns with traditional mentorship and instructional methods, incorporating respected community figures as educators

Abbreviations: FLX-MON = Flexibility–Monumentalism; NDPP = National Diabetes Prevention Program.

Adaptations were identified by the community through the Comprehensive Participatory Planning and Evaluation (CPPE) process [34, 35, 39, 40]; the framework used to organize and interpret them is from the first author’s dissertation [36].

Tailoring was prespecified at the curriculum level (cultural adaptation through the CPPE process) and permitted at the session level to respond to group preferences for recipes and workouts. Fidelity was monitored through (i) structured 30-min post-session debriefs with instructor-trainees and co-instructors that included review of content delivery, participant engagement, and any deviations from curriculum; and (ii) periodic session observation by the lead investigator. Detailed fidelity data will be reported in a subsequent process evaluation.

### Feasibility outcomes and engagement classification

Primary feasibility outcomes: (i) *enrollment,* number enrolled; (ii) *session attendance rate,* proportion of offered sessions attended per participant; (iii) *program retention,* proportion of attendance at the 4-month assessment.

A priori feasibility benchmarks for advancement to a Phase 2 hybrid effectiveness-implementation trial were: (i) 4-month retention ≥60% (exceeding the 52.6% published H/L NDPP retention benchmark) [21]; (ii) mean session attendance rate ≥50% of offered sessions, consistent with the minimum effective dose at which T2D risk reduction first becomes statistically significant [42]; and (iii) ≥ 50% of active participants achieving Tier 1 high-dose engagement.

To characterize engagement beyond binary attendance, participants were classified into three tiers: Tier 1 (high-dose): ≥50% of sessions (≥8 of 16, consistent with published H/L mean attendance [20] and effective doses [42]; Tier 2 (low-dose community-connected): <50% of sessions but attended session 16; and Tier 3 (discontinued): no program record at Session 16.

### Clinical outcomes

Cohort 1 outcomes were assessed at three timepoints: T1 baseline (July 1, 2025), T2 approximately 16 weeks (October 8–November 7, 2025), and T3 last available measurement (February–April 2026). One trainee was excluded from all outcomes due to initiation of a disease-modifying therapy (which can affect body weight) in November 2025 (retained in enrollment counts). Observational outcomes include body weight (kg), BMI (kg/m²), waist circumference (cm). HbA1c was not measured by the research team for this cohort; a subset of trainees voluntarily provided HbA1c values from their own clinical care, reported descriptively (see Results). Cohort 1 clinical outcomes are exploratory, as this cohort’s purpose was facilitator training rather than treatment.

Cohort 2 outcomes for 21 paired participants: body weight (kg), BMI (kg/m²), waist circumference (cm, Gulick tape), and HbA1c (%). All measures were conducted by the research team. Participants were classified as normal weight (BMI <25.0), overweight (25.0–29.9), or obese (≥30.0).

### Measures and data collection

All measures were collected by trained research staff at the community sites, in private rooms, using research-grade equipment and standardized research protocols. Body weight was measured in kilograms on calibrated Tanita WB-800H Plus scales, and height in centimeters with a stadiometer; body mass index was calculated as kg/m². Waist circumference was measured with a Gulick tape measure, and blood pressure assessed, per standard research protocols. For Cohort 2, HbA1c was measured using a point of care A1C device at the community site.

### Analysis

Descriptive statistics were calculated for each group. Clinical outcomes are reported as mean (SD) with percent change from baseline. No inferential testing was prespecified; this pilot was not powered for between-group comparisons.

Sample sizes for both cohorts were determined pragmatically rather than through formal power calculation, consistent with feasibility pilot designs intended to precede a powered hybrid effectiveness-implementation trial. Cohort 1 size (*n* = 18) was set by the number of community leaders identified through CAP partner networks who met eligibility criteria and committed to full-program completion within available funding; Cohort 2 size (*n* = 30 across two sites) was set by the group-delivery capacity of each community site (approximately 15-20 participants per site) within available funding. Implementation outcomes (enrollment, attendance, retention, tier classification) and intervention outcomes (weight, BMI, waist circumference, HbA1c) were analyzed separately: implementation outcomes are reported at the site and cohort level as proportions and means; intervention outcomes are reported for paired participants only, using complete-case analysis without imputation given the small sample. Adverse events, attrition reasons where available, and any mid-course adaptations to the intervention or implementation strategy were tracked throughout program delivery. No serious adverse events were reported or observed in either cohort; one Cohort 1 trainee initiated a disease-modifying therapy in November 2025 and was excluded from clinical outcomes (retained in enrollment and engagement counts). Because this therapy can affect weight but not attendance, she was excluded from all clinical outcomes yet retained in enrollment and engagement counts; removing a fully attending participant from the engagement denominators would itself bias those estimates.

## Results

### Cohort 1: CBO instructor–trainee cohort

#### Enrollment and characteristics

Eighteen community leaders enrolled as *Paso a Paso* instructor-trainees (all H/L, all female). Mean age was 48.8 (SD, 10.6) years (range 33–66; *n* = 16 with date of birth available). At baseline, mean weight was 70.9 (SD, 12.7) kg and mean BMI was 28.0 (SD, 5.0) kg/m² (*n* = 15): 5 (33%) normal weight, 3 (20%) overweight, and 7 (47%) obese. Mean waist circumference was 88.9 cm (SD, 11.4) (*n* = 17). Baseline characteristics are shown in Table 3.

**Table 3:** Instructor-trainee cohort: demographics and baseline characteristics (*n* = 18).

Characteristic	Overall *n* = 18
Age (years),^a^ (*n* = 16)	
Mean (SD)	48.8 (10.6)
Range	33–66
Ethnicity, *n* (%)	
Hispanic/Latino	18 (100)
Sex, *n* (%)	
Female	18 (100)
BMI category at baseline, *n* (%)^b^	
Normal weight (BMI <25.0)	5/16 (31.25)
Overweight (BMI 25.0–29.9)	3/15 (20)
Obese (BMI ≥30.0)	7/16 (43.8)
Baseline clinical measures, mean (SD), (*n* = 16)	
BMI (kg/m²)	28.0 (5.0)
Body weight (kg)	70.9 (12.7)
Waist circumference (cm), (*n* = 17)^c^	88.9 (11.4)

Abbreviation: BMI = body mass index.

a Age available for 16 of 18 enrolled trainees.

b BMI calculable for *n* = 16 of 18 trainees with baseline weight (excludes 1 trainee with no height and 1 with no baseline weight). One trainee excluded from all outcomes due to a disease-modifying therapy initiated in November 2025 (retained in *n* = 18).

c WC baseline *n* = 17; first available July–August 2025.

#### Session attendance and engagement

The 16 core sessions were delivered July 1–October 22, 2025. Mean sessions attended was 14.2 (SD, 2.6) [attendance rate 88.9% (SD, 16.0); median 93.8%; 31.2–100]. Seventeen of 18 trainees (94%) were Tier 1 high-dose, 1 (6%) was Tier 2 low-dose community-connected, and none (0%) were Tier 3 discontinued. All 18 were present at the final session. Attendance data are in Table 4.

**Table 4:** Session attendance and engagement tier: participate-to-lead (Cohort 1).

Measure	Overall *n* = 18
Session attendance 1–16 (July 1—October 22, 2025)	
In-person sessions offered	16
Attendance, mean (SD)	14.22 (2.6)
Attendance, median (range)	15 (5–16)
Attendance rate, mean (SD) (%)	88.9 (16.0)
Attendance rate, median (range) (%)	93.8 (31.2–100.0)
Engagement tier classification	
Tier 1. High-Dose (≥8 sessions), *n* (%)	17/18 (94)
Tier 2. Low-Dose, Community-Connected	1/18 (6)
(<8; present at final session), *n* (%)	
Tier 3. Discontinued (absent at final session), *n* (%)	0/18 (0)
4-month retention, *n* (%)	
Completed 4-month assessment	18/18 (100)
Eligible to become a *Paso a Paso* co-instructor (participate-to-lead), *n* (%)	18/18 (100)
Comparison: Published NDPP H/L benchmark	
Retention rate, (%)^a^	52.6
Session attended 7–8^b^	7–8
Effective dose^c^	≥7 sessions

Abbreviations: NDPP = National Diabetes Prevention Program; H/L = Hispanic/Latino.

a Cannon *et al.* [21] 18-week retention reported for Hispanic participants in a national cohort of in-person NDPP programs.

b Ritchie *et al.* [20] reported a mean attendance of 7.62 (SD, 7.17) sessions.

c Effective dose: the point at which T2D risk reduction first becomes statistically significant [42].

#### Exploratory clinical outcomes

Exploratory clinical outcomes for Cohort 1 were assessed at T1 (baseline), T2 (16 weeks; October 8–November 7, 2025), and T3 (last available measurement; February 1–April 10, 2026); baseline (T1) weights were a combination of research-measured and self-reported values, T2 (16-week) and T3 weights were research-measured in person. One trainee was excluded from all clinical outcomes due to initiation of a disease-modifying therapy for autoimmune condition in November 2025. At baseline, mean body weight was 70.9 (SD, 12.7) kg and mean BMI was 28.0 (SD, 5.0) kg/m² (*n* = 15), with 5 (33%) normal weight, 3 (20%) overweight, and 7 (47%) obese. At T2, mean weight change was −2.3% (SD, 5.6; *n* = 15), with 3 of 15 (20.0%) achieving ≥5% weight loss and 9 of 15 (60.0%) showing any weight loss; mean waist circumference declined by −2.7 cm (SD, 5.1). At T3, weight loss was more pronounced: mean change of −4.4% (SD, 6.0; *n* = 15), with 5 of 15 (33.3%) achieving ≥5% weight loss and mean waist circumference declining by −5.0 cm (SD, 8.3). Weight loss was greatest among trainees with obesity [T2: −5.4% (SD, 6.3) and T3: −8.3% (SD, 6.7)]. Full outcomes by BMI category are shown in Table 5. Cohort 1 clinical outcomes are exploratory. Separately, the mean CDC Prediabetes Risk Test score for the trainee cohort was 4.3, however, 5 fell within the prediabetic range (5.7%–6.4%) and 2 fell within the diabetic range (≥6.5%). Because these values were participant-provided rather than research-measured and represent a self-selected subset rather than a representative baseline, they are reported descriptively.

**Table 5:** Exploratory clinical outcomes at three timepoints by baseline BMI category: participate-to-lead (Cohort 1).

Outcome	T1: Baseline *n* = 16^a^	T2: 16 Weeks *n* = 15^b^	T3: Last Available *n* = 15^b^
Body weight			
Mean (SD), kg	70.9 (12.7) (*n* = 16)	70.8 (11.9) (*n* = 16)	69.0 (11.5) (*n* = 15)
% Change from T1, mean (SD)	—	−2.3 (5.6) (*n* = 15)	−4.4 (6.0) (*n* = 15)
≥5% weight loss from T1, *n* (%)	—	3 (20.0) (*n* = 15)	5 (33.3) (*n* = 15)
Any weight loss from T1, *n* (%)	—	9 (60.0) (*n* = 15)	10 (66.7) (*n* = 15)
BMI (kg/m²), *n* = 15^c^			
Mean (SD)	28.0 (5.0) (*n* = 15)	27.3 (4.8) (*n* = 15)	26.7 (4.6) (*n* = 15)
% Change from T1, mean (SD)	—	−2.35 (5.59)	−4.37 (5.99)
Body weight % change from T1, by BMI category			
Normal weight (BMI <25), *n* = 5	—	−0.8 (2.8) (*n* = 5)	−0.5 (2.4) (*n* = 5)
[T1 BMI = 21.90(1.64)]			
Overweight (BMI 25–29.9), *n* = 3	—	2.2 (3.4) (*n* = 3)	−1.7 (1.1) (*n* = 3)
[T1 BMI = 28.57(0.83)]			
Obese (BMI ≥30), *n* = 7	—	−5.4 (6.3) (*n* = 7)	−8.3 (6.7) (*n* = 7)
[T1 BMI = 32.77(1.87)]			
Waist circumference (cm)			
Mean (SD)	88.9 (11.4) (*n* = 17)	86.9 (9.5) (*n* = 16)	87.3 (8.6) (*n* = 12)
Change from T1, mean (SD)	—	−2.7 (5.1) (*n* = 16)	−5.0 (8.3) (*n* = 12)

Abbreviations: BMI = body mass index; SD = standard deviation.

Baseline (T1) weights were a combination of research-measured and self-reported values, T2 (16-week) and T3 weights were research-measured in person.

a T1 baseline *n* = 16 (*n* = 1 trainee excluded from all outcomes due to a disease-modifying therapy initiated in November 2025); *n* = 1 (no baseline weight).

b T2 window: October 8–November 7, 2025. T3 window: February 1–April 10, 2026.

c BMI *n* = 15 (excludes *n* = 1 no height and *n* = 1 no baseline weight).

#### Participate-to-lead program engagement and completion

All 18 completed the program, were eligible to be *Paso a Paso* instructors under RD mentorship and supervision, and six subsequently co-led sessions for Cohort 2 community cohorts. There were no discontinuations at 4 months. In a sensitivity analysis excluding the trainee who began a disease-modifying therapy, engagement was essentially unchanged: Tier 1 high-dose engagement was 16 of 17 (94.1%) versus 17 of 18 (94.4%) as reported, and 4-month retention remained 17 of 17 (100%).

### Cohort 2: community participants

#### Enrollment and characteristics

Thirty-three participants enrolled; three attended the orientation session only and are excluded from all analyses (active *n* = 30). All identified as Hispanic or Latino, 26 (86.7%) were female, mean age 48.9 (15.6) years (*n* = 29). Baseline measure data were available for 29 participants: mean BMI 31.7 (6.7) kg/m²; mean weight 77.3 (16.4) kg; mean waist circumference 95.8 (12.9) cm; mean HbA1c 5.6 (1.0%). At baseline: 3 (10.3%) normal weight, 9 (31.0%) overweight, 17 (58.6%) obese (Table 6).

**Table 6:** Participant demographics and baseline characteristics by site (Cohort 2).

Characteristic	Cohort 2a (Site A) *n* = 18	Cohort 2b (Site B) *n* = 12	Overall *n* = 30
Age (years),^a^ (*n* = 29)			
Mean (SD)	49.2 (15.4)	48.5 (16.6)	48.9 (15.6)
Range	21–77	27–74	21–77
Ethnicity, *n* (%)			
Hispanic/Latino	18 (100)	12 (100)	30 (100)
Sex, *n* (%)			
Female	17 (94.4)	9 (75.0)	26 (86.7)
Male	1 (5.6)	3 (25.0)	4 (13.3)
BMI category at baseline, *n* (%)			
Normal weight (BMI <25.0)	1 (5.6)	2 (18.2)	3 (10.3)
Overweight (BMI 25.0–29.9)	5 (27.8)	4 (36.4)	9 (31.0)
Obese (BMI ≥30.0)	12 (66.7)	5 (45.5)	17 (58.6)
Baseline clinical measures, mean (SD)			
BMI (kg/m²)^a^	31.9 (6.4) (*n* = 18)	31.2 (7.4) (*n* = 11)	31.7 (6.7) (*n* = 29)
Body weight (kg)	77.5 (16.9) (*n* = 18)	77.0 (16.3) (*n* = 11)	77.3 (16.4) (*n* = 29)
Waist circumference (cm)	94.1 (11.2) (*n* = 18)	98.6 (15.4) (*n* = 11)	95.8 (12.9) (*n* = 29)
HbA1c (%)	5.4 (0.3) (*n* = 18)	6.0 (1.6) (*n* = 11)	5.6 (1.0) (*n* = 29)

Abbreviations: BMI = body mass index; HbA1c = glycated hemoglobin; SD = standard deviation.

Three participants attended orientation only and are excluded from the active sample (*n* = 30).

Column headers report all active participants enrolled at each site. Age, BMI category, and baseline clinical measures are calculated on the subset with baseline data available (Cohort 2a *n* = 18, Cohort 2b *n* = 11, overall *n* = 29); percentages for these rows use those denominators.

a BMI calculated from measured height and weight (kg/m²).

#### Session attendance and engagement

Cohort 2a completed 15 sessions; Cohort 2b completed 13. Mean sessions attended was 7.6 (SD, 4.3), representing 53.6% (SD, 31.0%) of offered sessions. Twenty of 30 active participants (67%) achieved *high-dose* engagement: 11 of 18 at Cohort 2a (61%) and 9 of 12 at Cohort 2b (75%). Two Cohort 2a participants (11%) were Tier 2 *low-dose community-connected*; 8 (27%) were *discontinued*. Four-month retention was 73% (22 of 30 active participants): 14 of 18 at Cohort 2a (78%) and 8 of 12 at Cohort 2b (67%) (see Table 7).

**Table 7:** Session attendance and engagement tier by site (Cohort 2).

Measure	Cohort 2a (Site A) *n* = 18	Cohort 2b (Site B) *n* = 12	Overall *n* = 30
In-person sessions offered	15	13	—
Attendance, mean (SD)	7.0 (4.7)	7.5 (4.5)	7.2 (4.5)
Attendance, median (range)	8.0 (0–14)	9.0 (0–12)	8.0 (0–14)
Attendance rate, mean (SD) (%)^a^	46.7 (31.0)	57.7 (34.9)	53.6 (31.0)
Attendance rate, median (range) (%)^a^	53.3 (0.0–93.3)	69.2 (0.0–92.3)	61.5 (0.0–93.3)
Engagement tier classification, *n* (%)			
Tier 1. High-Dose (≥8 sessions), *n* (%)	11 (61)	9 (75)	20 (67)
Tier 2. Low-Dose, Community-Connected	2 (11)	0 (0)	2 (7)
(<8; present at final session), *n* (%)			
Tier 3. Discontinued (absent at final session), *n* (%)	5 (28)	3 (25)	8 (27)
4-month assessment retention, *n* (%)			
Completed 4-month assessment	14 (78%)	8 (67%)	22 (73%)

a Attendance rate calculations based on sessions offered (sessions attended ÷ sessions offered at their site).

A notable re-engagement pattern was observed at Cohort 2a: 9 of 18 participants absent during Sessions 8–10 (winter holiday period) returned at Session 11 at which point attendance rebounded to 67%. This re-engagement has not been documented in standard NDPP cohorts, where attendance loss in this period is typically permanent [20].

#### Four-month clinical outcomes

Outcomes for 21 paired participants are reported in Table 8 (by BMI category) and Table 9 (by site and BMI category). Overall, mean % body weight change was −1.5% (SD, 2.3) at 4 months, with 17 of 21 participants (81%) achieving any net weight reduction and 1 of 21 (5%) achieving ≥5% weight loss. Mean waist circumference declined by −2.0 (SD, 2.6) cm overall, with reductions observed across the overweight and obese subgroups (−1.3 cm and −2.8 cm, respectively). Mean HbA1c was stable at 4 months (baseline 5.6 ± 1.0% vs. 4-month 5.6 ± 0.9%), with a mean change of −0.9% (SD, 3.3) across the paired sample.

**Table 8:** Clinical outcomes at 4 months by baseline BMI category (Cohort 2).

Outcome	Normal weight BMI <25	Overweight BMI 25–29.9	Obese BMI ≥30	Overall
Body weight (kg)				
Baseline, mean (SD)	57.3 (2.7) (*n* = 3)	66.7 (11.5) (*n* = 9)	86.5 (13.2) (*n* = 17)	77.3 (16.4) (*n* = 29)
4-Month, mean (SD)	59.2 (0.4) (*n* = 2)	60.8 (6.6) (*n* = 7)	83.1 (13.8) (*n* = 12)	74.9 (17.0) (*n* = 22)
% Change, mean (SD)	1.0 (2.6) (*n* = 2)	−1.7 (1.5) (*n* = 7)	−1.9 (2.6) (*n* = 12)	−1.5 (2.3) (*n* = 21)
BMI (kg/m²)^a^				
Baseline, mean (SD)	21.7 (1.2) (*n* = 3)	27.2 (1.9) (*n* = 9)	35.8 (5.4) (*n* = 17)	31.7 (6.7) (*n* = 29)
4-Month, mean (SD)	22.5 (0.7) (*n* = 2)	26.7 (1.8) (*n* = 7)	35.5 (6.4) (*n* = 12)	31.6 (7.0) (*n* = 22)
% Change, mean (SD)	2.4 (3.4) (*n* = 2)	0.1 (4.5) (*n* = 7)	−1.7 (2.9) (*n* = 12)	−0.7 (3.6) (*n* = 21)
HbA1c (%)				
Baseline, mean (SD)	5.1 (0.2) (*n* = 3)	6.0 (1.7) (*n* = 9)	5.5 (0.3) (*n* = 17)	5.6 (1.0) (*n* = 29)
4-Month, mean (SD)	5.0 (0.3) (*n* = 2)	5.9 (1.5) (*n* = 7)	5.4 (0.2) (*n* = 12)	5.6 (0.9) (*n* = 22)
% Change, mean (SD)	0.0 (0.0) (*n* = 2)	−2.0 (4.6) (*n* = 7)	−0.4 (2.6) (*n* = 12)	−0.9 (3.3) (*n* = 21)
Waist circumference (cm)				
Baseline, mean (SD)	74.7 (4.1) (*n* = 3)	91.2 (7.1) (*n* = 9)	101.9 (11.3) (*n* = 17)	95.8 (12.9) (*n* = 29)
4-Month, mean (SD)	72.9 (2.5) (*n* = 2)	88.3 (5.9) (*n* = 7)	101.4 (11.0) (*n* = 12)	95.2 (13.2) (*n* = 22)
Change, mean (SD), cm	0.4 (0.1) (*n* = 2)	−1.3 (1.7) (*n* = 7)	−2.8 (3.0) (*n* = 12)	−2.0 (2.6) (*n* = 21)

Abbreviations: BMI = body mass index; HbA1c = glycated hemoglobin; SD = standard deviation.

% Change = ([4-month − baseline]/baseline) × 100.

a BMI calculated from measured height and weight (kg/m²).

**Table 9:** Clinical outcomes at 4 months by site and baseline BMI category (Cohort 2).

	Cohort 2a (Site A)				Cohort 2b (Site B)				Overall
	Normal *n* = 1	OW *n* = 4	Obese *n* = 8	Total *n* = 14	Normal *n* = 2	OW *n* = 4	Obese *n* = 5	Total *n* = 7	*n* = 21
% Change, Mean (SD)		−1.4 (1.6)	−2.9 (2.0)	−2.3 (1.9)	1.0 (2.6)	−2.3 (1.6)	1.0 (2.2)	0.1 (2.4)	−1.5 (2.3)
Waist circumference change, mean (SD), cm		−0.5 (1.2)	−3.5 (2.9)	−2.4 (2.8)	0.4 (0.1)	−3.4 (0.4)	−0.5 (2.2)	−1.1 (2.1)	−2.0 (2.6)

Abbreviation: OW = overweight.

% Change = ([4-month − baseline]/baseline) × 100.

Outcomes varied by BMI category and were most favorable in the obese subgroup, which accounted for 12 of 21 paired participants. Participants with obesity achieved a mean weight change of −1.9% (SD, 2.6), a mean waist circumference reduction of −2.8 (SD, 3.0) cm, and a mean HbA1c change of −0.4% (SD, 2.6). Participants in the overweight category showed a mean weight change of −1.7% (SD, 1.5) and a mean waist circumference reduction of −1.3 (SD, 1.7) cm. Normal-weight participants (*n* = 2 paired) showed a mean weight increase of +1.0% (SD, 2.6), consistent with expected stability at this BMI range.

Site-level differences were notable. At Cohort 2a (Site A), 14 paired participants achieved a mean weight change of −2.3% (SD, 1.9), with the obese subgroup (*n* = 8) showing the largest reduction at −2.9% (SD, 2.0) and a mean waist circumference decline of −3.5 (SD, 2.9) cm. At Cohort 2b (Site B), 7 paired participants showed a mean weight change of +0.1% (SD, 2.4), with the overweight subgroup (*n* = 4) at −2.3% (SD, 1.6) and the obese subgroup (*n* = 5) showing a mean weight change of +1.0% (SD, 2.2). Three high-dose (Tier 1) participants did not achieve weight reduction; non-response among adequately engaged participants may reflect biological heterogeneity and social determinants of health beyond program dose.

## Discussion

This pilot study examined the feasibility and preliminary clinical outcome trajectories of *Paso a Paso*, a community relevant weight management and diabetes prevention program co-designed with H/L community members across two community sites in Salt Lake County, Utah. Findings support the feasibility and early promise of *Paso a Paso,* with engagement rates exceeding published NDPP benchmarks for H/L participants and directionally favorable anthropometric trajectories, particularly among participants in the obesity subgroup.

### Engagement, attendance, and retention substantially exceed NDPP benchmarks

The 73%–100% *Paso a Paso* 4-month (16-session) retention rate compares favorably with national NDPP data, in which only 43% of all participants completed ≥16 core sessions overall [41]. This pattern is especially pronounced among H/L enrollees: in a single-system cohort study by Ritchie *et al.* [20], H/L participants attended a mean of 7.62 sessions compared with 10.36 for NHW participants (*P* < .01), and were approximately half as likely to attend at all (odds ratio, 0.52; 95% CI, 0.36–0.77). In a national analysis of 41 203 participants enrolled in CDC-recognized in-person lifestyle change programs [21], attrition was highest immediately after the first session and at weeks 17–18, coinciding with the transition from weekly to monthly sessions. H/L participants demonstrated the lowest retention of any racial or ethnic group across the program, with 52.6% retained through 18 weeks compared with 70.5% for NHW participants, a gap of nearly 18 percentage points that persisted throughout the program [21].

These retention outcomes reflect intentional design choices rather than chance. We attribute the elevated engagement and retention to the delivery by trusted bilingual RDN and community instructors; the multi-year community co-design (CPPE) that grounded the intervention content and strategies; culturally grounded adaptation strategies attuned to the domains of collectivism and monumentalism; community-site delivery; flexible session-level tailoring; and strategies mapped to the ERIC implementation taxonomy detailed in the Methods. The holiday-window re-engagement and the Tier 2 “community-connected” retention pattern are consistent with this community infrastructure.

Moreover, Ritchie *et al.* [20] found that once attendance was controlled for, weight loss differences by ethnicity were no longer statistically significant, suggesting that disparate outcomes were driven by differential engagement rather than by program efficacy or biological susceptibility. Additionally, offering Spanish-language sessions did not close the participation gap, attendance and weight loss outcomes were nearly identical between English- and Spanish-preferring Latino participants, underscoring the need for culturally tailored strategies that go beyond language accommodation to improve NDPP retention among H/L adults [20]. The H/L NDPP participation gap therefore appears structural rather than biological. *Paso a Paso’*s engagement and retention outcomes are consequently more than process metrics: they may represent the structural prerequisite for downstream clinical benefit.

A notable re-engagement pattern was observed at Cohort 2a, where 9 of 18 participants absent during the winter holiday period returned at Session 11. This type of re-engagement is not documented in standard NDPP cohorts, where holiday-window attrition is typically permanent [19, 20]. The Tier 2 *low-dose community-connected* classification (*n* = 2) participants attending fewer than 50% of sessions but retained at the 4-month assessment illustrates how *Paso a Paso’s* community infrastructure sustained program retention, and perhaps identification and belonging.

### Instructor–trainee engagement supports the participate-to-lead model

Cohort 1 demonstrated exceptional engagement: 100% retention, 94% *high-dose* attendance, 88.9% mean attendance, and zero discontinuations, in contrast to the significantly lower retention consistently documented for H/L participants in standard NDPP cohorts [19, 20] and the national data [41]. These results provide preliminary support for the community-expressed insight that shaped *Paso a Paso*: community leaders identified the need to experience the full program before leading it [34, 35]. Trainees who personally experienced the program likely developed an authentic connection to program content. One trainee, attending only 5 of 16 sessions but present at session 16 mirrors the same community retention pattern observed in the community cohort (Tier 2 *low-dose community-connected*). Because this two-cohort pilot was not designed to test whether instructors’ prior program completion improved participants’ engagement in subsequent cohorts, we interpret the participate-to-lead model as consistent with, and providing preliminary, hypothesis-generating support for, this approach rather than as validating it; a formal test is a prespecified aim of the planned hybrid effectiveness-implementation trial.

### Preliminary clinical outcomes and diabetes risk implications

Preliminary clinical outcomes at the 4-month core program milestone should be interpreted in context: Cohort 2 participants have eight monthly maintenance sessions remaining, and this pilot was not powered to detect statistically significant effects. However, the magnitude and pattern of observed changes in body weight and waist circumference warrant careful consideration against established benchmarks for diabetes risk reduction. Although the original Diabetes Prevention Program Randomized Controlled Trial (DPP) [13] demonstrated that a 5%–7% weight loss reduces T2D incidence by 58%, this magnitude of weight loss has been difficult to replicate at scale and in real-world settings [19, 20, 41]. Therefore, the clinical significance of observed changes in body weight and waist circumference in *Paso a Paso* should not be understated. Weight loss remains the dominant predictor of reduced diabetes incidence: for *every kilogram of weight lost*, there is an approximately 16% reduction in diabetes risk, adjusted for changes in diet and physical activity [43]. Placing *Paso a Paso’s* observed mean weight loss of −2.3% (SD 1.9) in the Cohort 2a high-dose subgroup within this dose-response trajectory suggests a clinically meaningful reduction in diabetes risk, independent of attainment of the 5% threshold. In addition, waist circumference measures provide prognostic information beyond body weight alone. Within the original DPP, the lifestyle intervention reduced waist circumference by 7.5% in men and 6.1% in women, with concurrent reductions in visceral fat [44]. *Paso a Paso’s* 4-month reductions in waist circumference across all groups, represent a clinically meaningful intermediate outcome on the causal pathway to type 2 diabetes risk reduction, supporting that the program is producing biologically relevant changes even among participants who did not achieve the 5% weight loss threshold.

The Cohort 1 instructor-trainees achieved clinically meaningful outcomes over a 9-month arc: −2.35% (SD, 5.59) at 16 weeks and −4.37% (SD, 5.99) at end of program, with 33% achieving ≥5% weight loss. The subgroup of participants with obesity had a mean weight loss of −8.3% (SD, 6.7), exceeding the mean of 4.2% reported among NDPP participants [41]. These outcomes carry dual significance: they are consistent with program efficacy in a high-engagement population, though exploratory and hypothesis-generating rather than confirmatory, and reflect what instructors personally experienced before delivering the program, a form of embodied credibility that conventional community health worker (CHW) training cannot replicate. As exploratory findings from a small, single-arm feasibility cohort, these results require confirmation in an adequately powered trial.

### Contextual factors: holiday season, funding constraints, and site differences

A 3-month Clinical and Translational Science Institute (CTSI) funding delay compressed delivery to 15 sessions at Cohort 2a and 13 at Cohort 2b, versus the planned 16 for each site. Despite this structural constraint, both community cohorts completed 4-month assessments in March 2026, a timeline that spanned the peak U.S. holiday season in its entirety. Unlike programs that begin in lower-risk months and subsequently navigate the holiday window with established behavioral routines, both Cohort 2a and Cohort 2b initiated lifestyle change during the holiday season itself. Cohort 2a enrolled in November, at the onset of a period during which even participants in active weight-loss programs consistently gain 0.4–0.9 kg [45], while Cohort 2b enrolled in December, at the peak of the holiday season that alone accounts for a mean holiday gain of 0.48 kg (not fully reversed) in U.S. adults [46] and during which individuals with obesity gain disproportionately more than lean peers [45]. Participants in both cohorts therefore had no opportunity to establish behavioral momentum before encountering the highest-risk period for weight gain. That 81% of paired participants nonetheless achieved net body weight reductions is an exploratory observation from a small single-arm sample that should be interpreted cautiously; it is consistent with, but cannot establish, a protective role of Paso a Paso’s community infrastructure against the environmental pressures that typically disrupt weight management during this period.

Site-level differences further contextualize the outcomes. The waist circumference reduction observed in the Cohort 2a subgroup of participants with obesity, −3.5 cm (SD, 2.9 cm) at 4 months, approaches the lower bound of individual Medical Nutrition Therapy (MNT) benchmarks for clinically meaningful waist circumference (WC) reduction of −3.8–5.9 cm [47, 48], that this benchmark was achieved in a group-based community setting is notable: a single registered dietitian working alongside a trained community instructor can reach 15–20 participants per cohort, representing a particularly meaningful scalability advantage for H/L communities where access to individualized MNT remains limited.

### Limitations

This study has several limitations. (i) This was a single-arm feasibility pilot without a control group. (ii) All clinical analyses are descriptive; the paired outcome sample (*n* = 21) is insufficient for inferential testing. (iii) Site timing differences cannot be fully disentangled from site-level characteristics. (iv) Session delivery for Cohort 2 fell below the planned dose. (v) In Cohort 1, baseline body weight combined self-reported and research-measured values, whereas T2 and T3 were research-measured in person; Cohort 1 clinical outcomes are therefore reported as exploratory. (vi) For Cohort 1, the analytic sample is *n* = 15 with paired T1→T3 weight data; one trainee was excluded due to medical confound; two contributed partial data only. (vii) The absence of systematic baseline HbA1c collection in the instructor cohort is a limitation. (viii) The 9-month Cohort 1 assessment window versus 4-month Cohort 2 window limits direct cross-cohort comparisons. (ix) All instructor-trainees were female community representatives, limiting generalizability of the instructor training model, though female predominance is common in lifestyle studies. (x) The design cannot causally link instructors’ prior program completion to participant engagement in subsequent cohorts; this was not a prespecified test and is reserved for the planned powered trial. (xi) Cohort 1 trainees received per-session compensation; although no payment was contingent on weight, HbA1c, attendance thresholds, or program completion, per-session compensation may have contributed to this cohort’s high attendance.

## Conclusions

*Paso a Paso* suggests feasibility for engaging H/L adults in a community-based diabetes prevention program, with engagement and retention substantially exceeding NDPP benchmarks. The participate-to-lead instructor training model, in which community leaders complete the full program before facilitating it, produced 100% retention, a median attendance rate of 93.8%, and clinically meaningful weight and WC reductions, providing preliminary, hypothesis-generating support for the community-expressed implementation strategy. The three-tier engagement framework captures program connections that binary attendance metrics miss. Together, these findings support advancement to a powered hybrid effectiveness-implementation trial, with 12-month co-primary outcomes of ≥5% weight loss and HbA1c, and WC as a key secondary outcome tailored to the diabetes and cardiometabolic risk profile of H/L participants.

## Data Availability

De-identified attendance data are available from the corresponding author upon reasonable request. Clinical data are maintained in REDCap systems and are subject to IRB data sharing protocols.

## Welfare of animals

This article does not contain any studies with animals performed by any of the authors.

## Additional information

Information on previous presentation of the information reported in the manuscript: Abstracts were presented as posters at the 2024 American Heart Association, 2024 Society of General Internal Medicine Annual Meeting, Oral presentation at Utah Public Health Conference: Past, Present, Future: Honoring the past, navigating the present, and designing the future of public health, and 2026 Society of General Internal Medicine Annual Meeting.
